# Supplementary material for: Structural basis of a redox-dependent conformational switch that regulates the stress kinase p38α
Source: Nat Commun. 2023 Dec 1;14:7920. doi: 10.1038/s41467-023-43763-5 (PMC10692146; doi:10.1038/s41467-023-43763-5)
Supplement: Supplementary file 1 — Supplementary Information [file 41467_2023_43763_MOESM1_ESM.pdf]

Supplementary information for

Structural basis of a redox-dependent conformational switch that regulates the stress kinase p38α

Joan Pous[1,7], Blazej Baginski[1,5,7], Pau Martin-Malpartida[1,7], Lorena González[1,6], Margherita Scarpa[1], Eric Aragon[1], Lidia Ruiz[1], Rebeca A. Mees[1], Javier Iglesias-Fernández[2], Modesto Orozco[1,3], Angel R. Nebreda[1,4,8] and Maria J. Macias[1,4,8]

[1] Institute for Research in Biomedicine (IRB Barcelona), The Barcelona Institute of Science and Technology, Baldiri Reixac, 10, 08028 Barcelona, Spain.

[2] Nostrum Biodiscovery, Josep Tarradellas 8-10, 3-2, 08029 Barcelona, Spain

[3] Departament de Bioquímica i Biomedicina, Facultat de Biologia, Universitat de Barcelona, 08028 Barcelona, Spain.

[4] Institució Catalana de Recerca i Estudis Avançats (ICREA), Passeig Lluís Companys 23, 08010 Barcelona, Spain.

[5] Present address: Global Health Medicines R&D, GSK, c/ Severo Ochoa, 2, 28760, Tres Cantos, Madrid, Spain

[6] Present address: Grupo Menarini España, c/ d'Alfons XII, 587, 08918 Badalona, Barcelona, Spain

[7] These authors contributed equally: Joan Pous, Blazej Baginski, Pau Martin-Malpartida

[8] These authors jointly supervised this work: Angel R. Nebreda, Maria J. Macias. e-mail: angel.nebreda@irbbarcelona.org; maria.macias@irbbarcelona.org

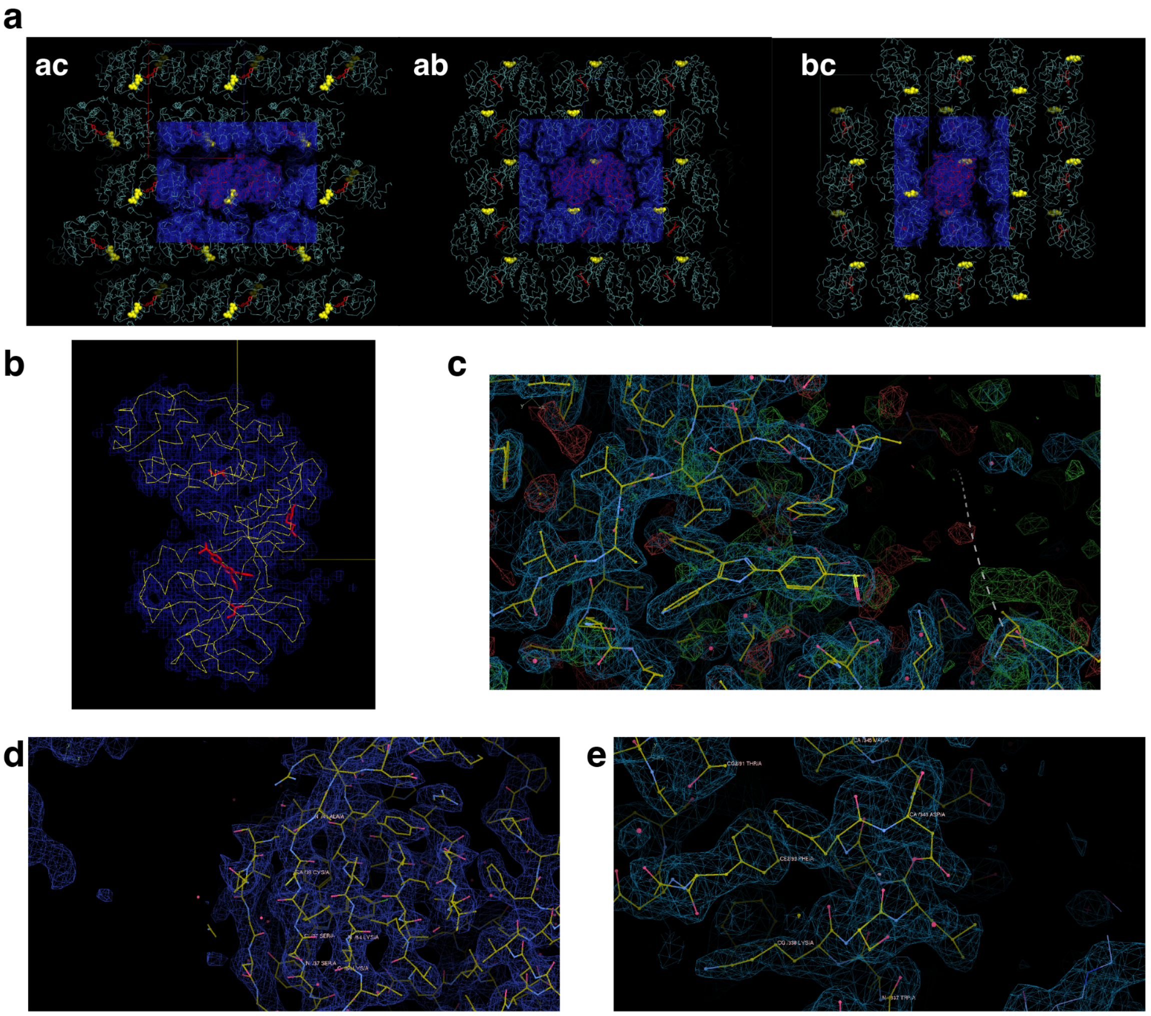

Supplementary Fig. 1. Lattice and Asymmetric unit.

a. Molecule: The two structures determined in this work have identical ASU. Ribbon representation of the $P2_12_12_1$ lattice for the 8ACO structure. Symmetry neighbors are shown in cyan, the asymmetric Unit (ASU) is shown in red. Cys119 and Cys162 (those defining the disulfide bond) are colored as yellow spheres, whereas the SB203580 molecule is displayed as red sticks. 2mFo-DFc density in blue was calculated with CCP4's FFT using coefficients from Refmac5 (F1=FWT PHI=PHWT) and displayed at a density of 1σ and a block of 20Å around the ASU. Map slab adjusted to ASU depth. Left: plane ac. Center: plane ab. Right: plane bc. Third axis pointing up in all three figures. Figures were generated with Pymol. **b.** Ribbon representation of the Asymmetric Unit of 8ACO in yellow. SB203580 ligand and Cys residues are shown as red sticks. Map parameters as in **a**. The view is oriented as in the middle figure above, but with c rotated by 90 degrees. **c.** Snapshots of the electron density. Maps automatically calculated from 8ACM reflections. Blue: FWT, PHIWT map at 1σ

density. DELFWT (green) PHDELWT (red) difference maps at 2σ and -2σ density respectively. The displayed area is around the SB203580 ligand with the Tyr35 above and the disordered A-loop to the right (white dots). The lack of difference map prevented to trace the A-loop. Figures generated with Coot. **d.** 8ACM density details. The 5-strand beta sheet of the N-lobe. Blue maps calculated as in **c**. **e.** Residues 337-345 of the C-terminal helix and surrounding area.

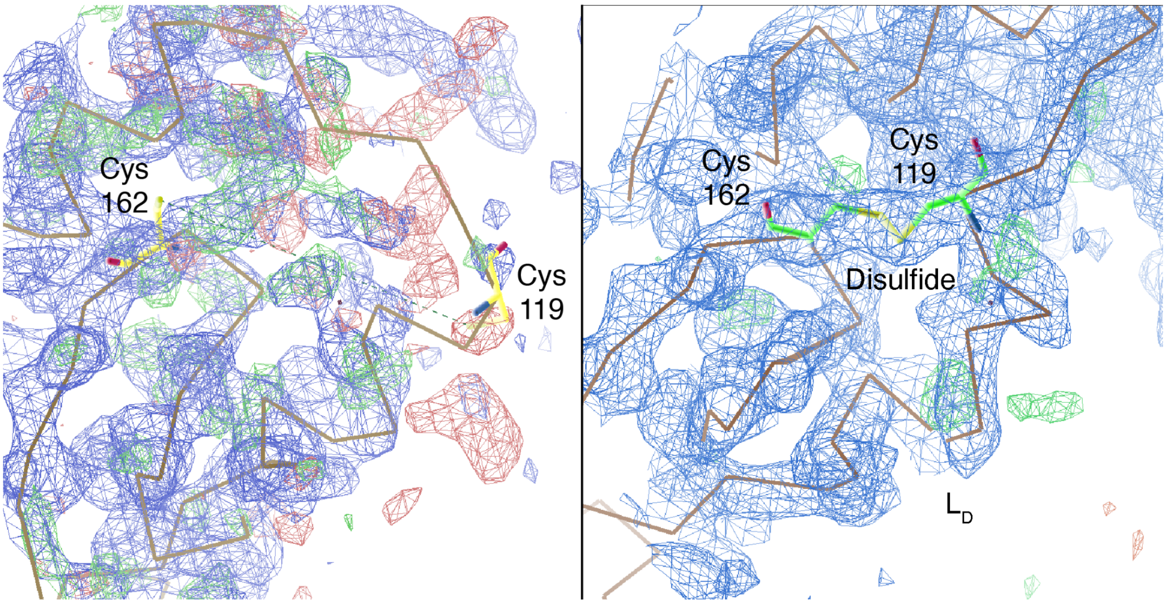

Supplementary Fig. 2. **Density directly after the molecular replacement and after refinement.**

Electron density map plotted at 1σ showing the region around Cys119 and Cys162 residues. Left panel shows the electronic density and the reduced 4LOO structure, directly after molecular replacement. The 4LOO structure does not fit the density. Right panel is the final, refined 8ACM structure that fits the electronic density. The intramolecular Cys119-Cys162 disulfide bond and the presence of the L_D loop are indicated.

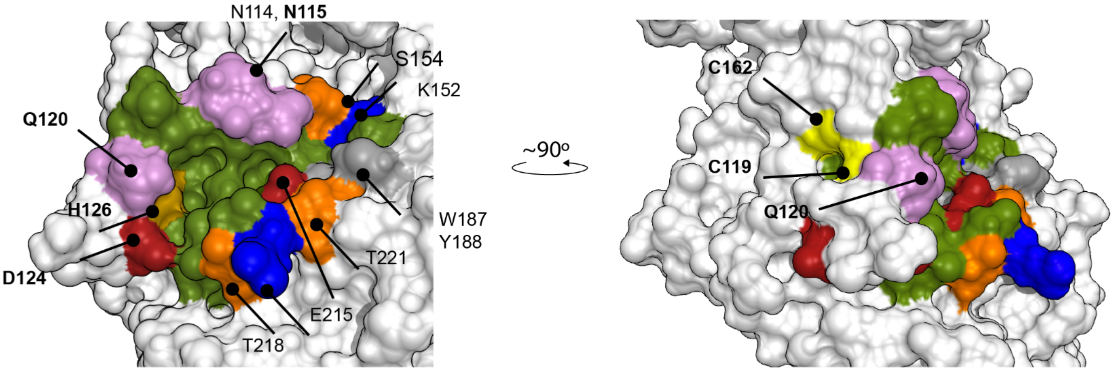

Supplementary Fig. 3. **Two views of the binding cavity observed in the oxidized form.** The disulfide bond (yellow) is located behind the cavity and it is not visible in the orientation shown in the left panel. Color code as in Fig 3c.

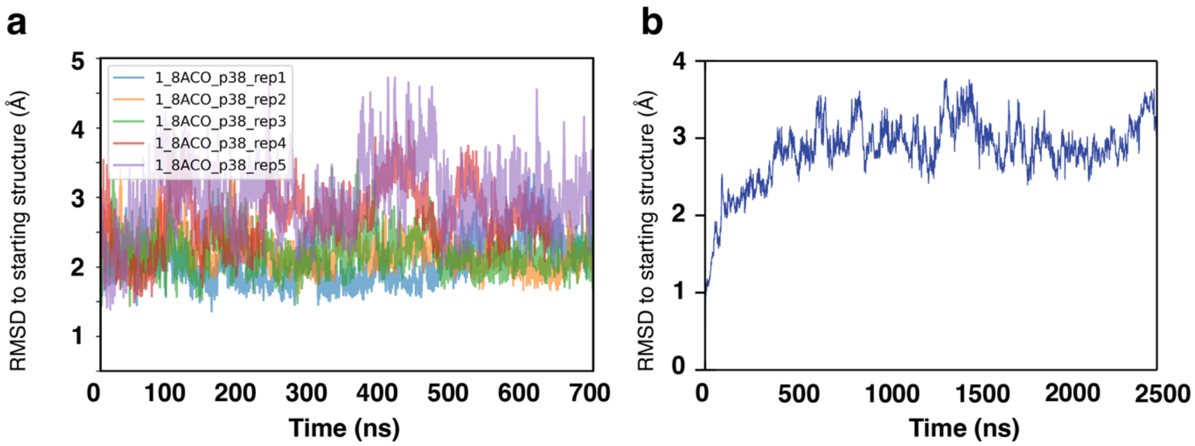

Supplementary Fig. 4. **RMSD analysis.**

a. RMSD evolution during the Molecular Dynamics (MD) simulations. The starting model was the oxidized form (PDB: 8ACO) with the disulfide bond open, and maintaining Cys119 and Cys162 as close as 3Å. Five replicates of 700 ns were performed **b.** RMSD evolution during the Gaussian accelerated MD (GaMD) run for 2500 ns. In this case, the starting structure was the reduced p38α structure (PDB: 5R8W).

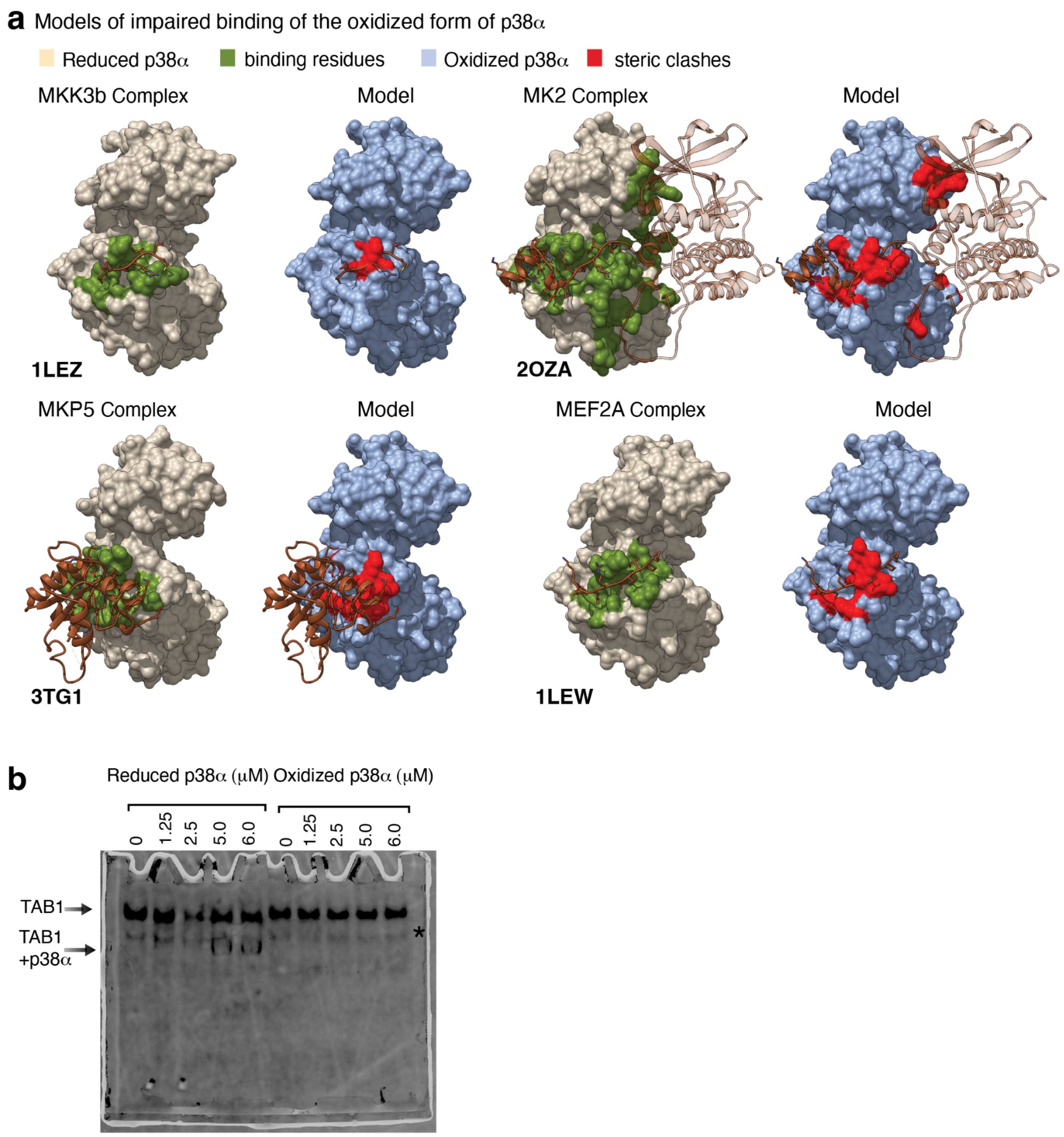

Supplementary Fig. 5. Models of the impaired binding of regulators and substrates to the closed conformation of p38α.

a. Structures of complexes of the indicated ligands with the reduced form of p38α, with the PDB entry shown next to each structure. The corresponding model using the oxidized form of p38α is shown on the right, with the surface displayed in light blue to distinguish structures from models. In both cases, the ligand is displayed as in the original complexes. Whereas in the complexes with reduced p38α the ligand is accommodated in the binding cavity (green), the conformational rearrangements of the oxidized p38α hamper its interaction with large parts of the ligands. Clashes in models are shown in red. Supplementary Movie 2 and Supplementary Movie 3 illustrate how the oxidized form interferes with ligand binding. **b.**

Independent replicate of the Electrophoretic mobility shift assay (EMSA) analysis shown in Fig. 5b. Oxidized and reduced p38α proteins were incubated at the indicated concentrations with the fluorescently labeled TAB1 peptide (0.25 μM in buffer with BSA 8 μg/mL). The asterisk indicates a non-specific interaction with BSA.

Supplementary Table 1. Setup for the Molecular dynamics (MD) and for the Gaussian accelerated MD (GaMD) simulations.

	**MD simulation**	**GaMD simulation**
**Starting structure**	Oxidized 8ACO	Reduced 5R8W
**Box dimensions**	Octahedron<br>x,y,z= 91.3304 Å	Truncated Octahedron<br>x,y,z= 86.0557 Å
**Total number of atoms**	51922	43952
**Water molecules**	15437	12760
**Ions**	7 Na+ (20 mM)	27 Na+ (91mM)<br>20 Cl- (67mM)
**Simulation time (nanoseconds)**	500	2500
