# Supplementary material for: Structural basis of a redox-dependent conformational switch that regulates the stress kinase p38α
Source: Nat Commun. 2023 Dec 1;14:7920. doi: 10.1038/s41467-023-43763-5 (PMC10692146; doi:10.1038/s41467-023-43763-5)
Supplement: Supplementary file 3 — Description of Additional Supplementary Files [file 41467_2023_43763_MOESM3_ESM.pdf]

Description of Additional Supplementary Files

File Name: Supplementary Movie 1
Description: Animation showing the transition between the reduced (PDB:3OBG) and the oxidized (PDB:8ACM) p38α structures. αD/LD is shown in gold, A-loop in purple, disulfide bridge in yellow.

File Name: Supplementary Movie 2
Description: Animation showing the transition between the reduced p38α bound to MKK3B (PDB:1LEZ) and the oxidized p38α (PDB:8ACM). Clashes between the MKK3B peptide and the oxidized p38α are shown in red.

File Name: Supplementary Movie 3
Description: Animation showing the transition between the reduced p38α bound to TAB1 (PDB:4LOO) and the oxidized p38α (PDB:8ACM). Clashes between the TAB1 peptide and the oxidized p38α are shown in red.
